# Supplementary material for: P-TRAP: a Panicle Trait Phenotyping tool
Source: BMC Plant Biol. 2013 Aug 29;13:122. doi: 10.1186/1471-2229-13-122 (PMC3848748; doi:10.1186/1471-2229-13-122)
Supplement: Additional file 7 — P-TRAP source code. The Netbeans project that contains the source code of the application. [file 1471-2229-13-122-S7.zip › javadoc.zip!/javadoc/org-netbeans-modules-riceoptions/org/netbeans/modules/riceoptions/RiceOptionsOptionsPanelController.html]

RiceOptionsOptionsPanelController (Rice Options)


JavaScript is disabled on your browser.


- Package
- Class
- Tree
- Deprecated
- Index
- Help

*org.netbeans.modules.riceoptions 1.0*

- Prev Class
- Next Class

- Frames
- No Frames

- All Classes

- Summary:
- Nested |
- Field |
- Constr |
- Method

- Detail:
- Field |
- Constr |
- Method


org.netbeans.modules.riceoptions

## Class RiceOptionsOptionsPanelController

- java.lang.Object
- - org.netbeans.spi.options.OptionsPanelController
  - - org.netbeans.modules.riceoptions.RiceOptionsOptionsPanelController

- ---

    

  ```
  public final class RiceOptionsOptionsPanelController
  extends org.netbeans.spi.options.OptionsPanelController
  ```

  The handler of the rice options dialog.

  Author:
  :   faroqal-tam

- - ### Nested Class Summary

    - ### Nested classes/interfaces inherited from class org.netbeans.spi.options.OptionsPanelController

      `org.netbeans.spi.options.OptionsPanelController.ContainerRegistration, org.netbeans.spi.options.OptionsPanelController.Keywords, org.netbeans.spi.options.OptionsPanelController.KeywordsRegistration, org.netbeans.spi.options.OptionsPanelController.SubRegistration, org.netbeans.spi.options.OptionsPanelController.TopLevelRegistration`
  - ### Field Summary

    - ### Fields inherited from class org.netbeans.spi.options.OptionsPanelController

      `PROP_CHANGED, PROP_HELP_CTX, PROP_VALID`
  - ### Constructor Summary

    Constructors

    Constructor and Description

    `RiceOptionsOptionsPanelController()`
  - ### Method Summary

    Methods

    | Modifier and Type | Method and Description |
    | --- | --- |
    | `void` | `addPropertyChangeListener(java.beans.PropertyChangeListener l)` |
    | `void` | `applyChanges()` |
    | `void` | `cancel()` |
    | `javax.swing.JComponent` | `getComponent(org.openide.util.Lookup masterLookup)` |
    | `org.openide.util.HelpCtx` | `getHelpCtx()` |
    | `boolean` | `isChanged()` |
    | `boolean` | `isValid()` |
    | `void` | `removePropertyChangeListener(java.beans.PropertyChangeListener l)` |
    | `void` | `update()` |

    - ### Methods inherited from class org.netbeans.spi.options.OptionsPanelController

      `createAdvanced, getLookup, handleSuccessfulSearch, setCurrentSubcategory`
    - ### Methods inherited from class java.lang.Object

      `clone, equals, finalize, getClass, hashCode, notify, notifyAll, toString, wait, wait, wait`

- - ### Constructor Detail


    - #### RiceOptionsOptionsPanelController

      ```
      public RiceOptionsOptionsPanelController()
      ```
  - ### Method Detail


    - #### update

      ```
      public void update()
      ```

      **Specified by:**
      :   `update` in class `org.netbeans.spi.options.OptionsPanelController`


    - #### applyChanges

      ```
      public void applyChanges()
      ```

      **Specified by:**
      :   `applyChanges` in class `org.netbeans.spi.options.OptionsPanelController`


    - #### cancel

      ```
      public void cancel()
      ```

      **Specified by:**
      :   `cancel` in class `org.netbeans.spi.options.OptionsPanelController`


    - #### isValid

      ```
      public boolean isValid()
      ```

      **Specified by:**
      :   `isValid` in class `org.netbeans.spi.options.OptionsPanelController`


    - #### isChanged

      ```
      public boolean isChanged()
      ```

      **Specified by:**
      :   `isChanged` in class `org.netbeans.spi.options.OptionsPanelController`


    - #### getHelpCtx

      ```
      public org.openide.util.HelpCtx getHelpCtx()
      ```

      **Specified by:**
      :   `getHelpCtx` in class `org.netbeans.spi.options.OptionsPanelController`


    - #### getComponent

      ```
      public javax.swing.JComponent getComponent(org.openide.util.Lookup masterLookup)
      ```

      **Specified by:**
      :   `getComponent` in class `org.netbeans.spi.options.OptionsPanelController`


    - #### addPropertyChangeListener

      ```
      public void addPropertyChangeListener(java.beans.PropertyChangeListener l)
      ```

      **Specified by:**
      :   `addPropertyChangeListener` in class `org.netbeans.spi.options.OptionsPanelController`


    - #### removePropertyChangeListener

      ```
      public void removePropertyChangeListener(java.beans.PropertyChangeListener l)
      ```

      **Specified by:**
      :   `removePropertyChangeListener` in class `org.netbeans.spi.options.OptionsPanelController`


- Package
- Class
- Tree
- Deprecated
- Index
- Help

*org.netbeans.modules.riceoptions 1.0*

- Prev Class
- Next Class

- Frames
- No Frames

- All Classes

- Summary:
- Nested |
- Field |
- Constr |
- Method

- Detail:
- Field |
- Constr |
- Method
